# A novel organic-rich meteoritic clast from the outer solar system

**DOI:** 10.1038/s41598-019-39357-1

**Published:** 2019-02-28

**Authors:** Yoko Kebukawa, Motoo Ito, Michael E. Zolensky, Richard C. Greenwood, Zia Rahman, Hiroki Suga, Aiko Nakato, Queenie H. S. Chan, Marc Fries, Yasuo Takeichi, Yoshio Takahashi, Kazuhiko Mase, Kensei Kobayashi

**Affiliations:** 1 Faculty of Engineering, Yokohama National University, 79-5 Tokiwadai, Hodogaya-ku, Yokohama 240-8501 Japan; 2 Kochi Institute for Core Sample Research, JAMSTEC, B200 Monobe, Nankoku, Kochi 783-8502 Japan; 3 ARES, NASA Johnson Space Center, 2101 NASA Parkway, Houston, TX 77058 USA; 4 Planetary and Space Sciences, The Open University, Walton Hall, Milton Keynes, MK7 6AA United Kingdom; 5 Jacobs, NASA Johnson Space Center, Houston, TX 77058 USA; 6 Department of Earth and Planetary Systems Science, Hiroshima University, Kagamiyama, Higashi-Hiroshima, Hiroshima, 739-8526 Japan; 7 Institute of Space and Astronautical Science (ISAS), Japan Aerospace Exploration Agency (JAXA), 3-1-1 Yoshinodai, Sagamihara, 252-5210 Japan; 8 Institute of Materials Structure Science, High-Energy Accelerator Research Organization (KEK), 1-1 Oho, Tsukuba, Ibaraki 305-0801 Japan; 9 Department of Earth and Planetary Science, The University of Tokyo, Hongo, Bunkyo-ku, Tokyo, 113-0033 Japan; 10 Present Address: Department of Earth and Planetary Science, The University of Tokyo, Hongo, Bunkyo-ku, Tokyo, 113-0033 Japan; 11 Present Address: Department of Physical Sciences, The Open University, Walton Hall, Milton Keynes, MK7 6AA UK

## Abstract

The Zag meteorite which is a thermally-metamorphosed H ordinary chondrite contains a primitive xenolithic clast that was accreted to the parent asteroid after metamorphism. The cm-sized clast contains abundant large organic grains or aggregates up to 20 μm in phyllosilicate-rich matrix. Here we report organic and isotope analyses of a large (~10 μm) OM aggregate in the Zag clast. The X-ray micro-spectroscopic technique revealed that the OM aggregate has *sp*^2^ dominated hydrocarbon networks with a lower abundance of heteroatoms than in IOM from primitive (CI,CM,CR) carbonaceous chondrites, and thus it is distinguished from most of the OM in carbonaceous meteorites. The OM aggregate has high D/H and ^15^N/^14^N ratios (δD = 2,370 ± 74‰ and δ^15^N = 696 ± 100‰), suggesting that it originated in a very cold environment such as the interstellar medium or outer region of the solar nebula, while the OM is embedded in carbonate-bearing matrix resulting from aqueous activities. Thus, the high D/H ratio must have been preserved during the extensive late-stage aqueous processing. It indicates that both the OM precursors and the water had high D/H ratios. Combined with ^16^O-poor nature of the clast, the OM aggregate and the clast are unique among known chondrite groups. We further propose that the clast possibly originated from D/P type asteroids or trans-Neptunian Objects.

## Introduction

Xenolithic clasts are often found in a wide variety of meteorite groups^1–9^, some of which contain exotic organic matter (OM)^10^. Xenolithic clasts have been protected in host meteorites that are typically more metamorphosed and thus are physically strengthened by thermal annealing via heating processes occurring prior to the incorporation of the clasts. Hence, such clasts can contain primitive and fragile materials that would not have survived parent body alteration processes and atmospheric entry. The Zag meteorite is a H3-6 chondrite which fell in Morocco on August 1998, and is known to contain xenolithic, fluid inclusion-bearing halite crystals and a centimeter-sized carbonaceous chondrite-like clast^1,11^. The clast in the Zag meteorite consists of saponite, serpentine, Ca-Fe-Mg carbonates, Fe-Ni sulfides, magnetite, halite, minor olivine and pyroxene, as well as abundant large OM grains or aggregates up to 20 μm, indicating that the Zag clast has been subjected to significant aqueous alteration^12^.

We analyzed the molecular structure and isotope chemistry of a focused ion beam (FIB) section obtained from an OM aggregate using scanning transmission X-ray microscopy (STXM) coupled with X-ray absorption near edge structure (XANES) and nanoscale secondary ion mass spectrometry (NanoSIMS), as well as bulk O-isotopic analyses.

## Results

**Figure 1:**
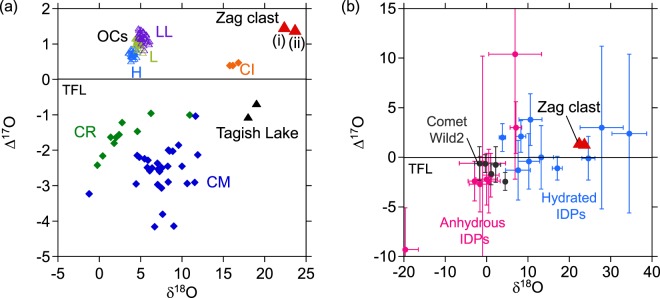
Oxygen isotope composition of the Zag clast shown in relation to various chondrite groups (**a**), IDPs and cometary dust particles (**b**). Ordinary chondrite (H, L, LL) data from Clayton *et al*.^55^, Carbonaceous chondrite (CI, CM, CR) data from Clayton and Mayeda^56^, Tagish Lake data from Brown *et al*.^44^, comet Wild2 particle data from McKeegan *et al*.^57^, IDP data from Aléon *et al*.^58^. Zag clast (i): this study, Zag clast (ii): analysis of the same clast reported by Zolensky *et al*.^13^. TFL: terrestrial fractionation line. The errors are smaller than the symbol sizes.

The oxygen isotopic composition of the clast is unique (δ^17^O = +13.13 ± 0.13‰; δ^18^O = +22.38 ± 0.17‰; ∆^17^O = +1.49 ± 0.04‰), plotting well away from the H chondrite field (Fig. 1). The bulk oxygen isotopic composition of the clast is somewhat related to the chondrites but yet clearly distinguished from them (Fig. 1a, Supplementary Fig. 2). The oxygen isotopic composition of the clast falls within the range of hydrated IDPs, although the oxygen isotopic compositions of the hydrated IDPs are highly varied (Fig. 1b). A similar isotopic composition of the Zag clast (different aliquot from the same clast) has been reported by Zolensky *et al*.^13^ (Fig. 1).

**Figure 2:**
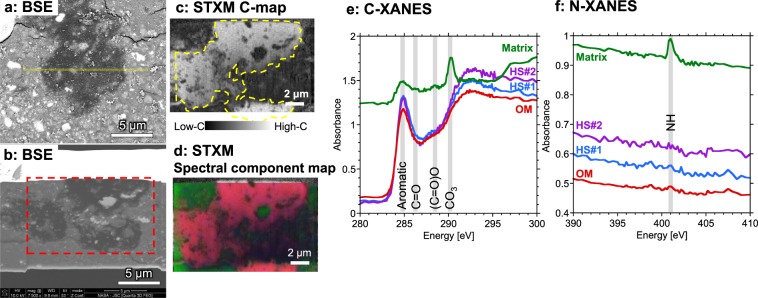
Scanning transmission X-ray microscopy (STXM) analyses of a focused ion beam (FIB) section containing the organic matter (OM) aggregate in the Zag clast. (**a**) Backscattered electron (BSE) image of a polished thin section of the organic aggregate (dark) in the carbonaceous clast in the Zag meteorite. FIB section was subsampled from the yellow region. (**b**) BSE image of the FIB section. STXM maps (**c**,**d**) were obtained from the location indicated by the red dotted line. (**c**) Carbon-map (at 292 eV) indicates the section is dominated by carbon. The OM aggregate is indicated by the yellow dotted line. (**d**) Spectral component map derived from C-XANES of OM (red) and matrix (green). The red and green regions correspond to the OM and matrix C-XANES spectra shown in (**e**). (**e**) The C-XANES of OM aggregate revealed that it is dominated by *sp*^2^ carbon (284.8 eV) while in the surrounding matrix carbon is mainly found as carbonates (290.3 eV) with some OM at 286.3 eV that is assigned to ketone (C=O) and 288.5 eV that is assigned to carboxyl/ester [(C=O)O]. (**f**) The OM aggregate does not show detectable N-XANES features while matrix shows a peak at 401.0 eV which is assigned to amines. The C- and N-XANES obtained from isotope hot spots (HS, see Fig. 3) are also shown.

The FIB section obtained from the OM aggregate showed a large carbon-dominated area over 10 μm in width that corresponded to the OM aggregate (Fig. 2a,b). The C-, N-XANES spectra of the OM aggregate (Fig. 2e,f) revealed that its molecular structure was *sp*^2^ (aromatic/olefinic) carbon dominated with few other functional groups, but the aromatic domains are small. The matrix contains carbonates and smaller amounts of OM containing other functional groups such as ketone, carboxyl/ester and possibly amine. A C-XANES spectrum of the OM aggregate showed a peak at 284.8 eV that is assigned to *sp*^2^ (aromatic/olefinic) carbon (Fig. 2d,e in red). The surrounding matrix area showed a peak at 290.3 eV that is assigned to carbonates (CO_3_) with some organic features at 284.8 eV, 286.3 eV (assigned to ketone [C=O]) and 288.5 eV (assigned to carboxyl/ester [(C=O)O]) (Fig. 2d,e in green). The C-XANES spectrum of the OM aggregate does not show other peaks that are characteristic of insoluble organic matter (IOM) in primitive chondrites (e.g., C=O and (C=O)O indicating primitive OM in Murchison meteorite)^14^, nor that in the thermally-metamorphosed meteorites (e.g., 1s-σ* exciton at 291.7 eV indicating graphene structures in the Allende meteorite)^15^. The C-XANES indicated that the OM aggregate was highly aromatic but aromatic domains were disordered and small. It should be noted that this does not mean that there is a complete absence of aliphatic, and O- and N-bearing functional groups, since small peaks could be a part of the unresolved continuous absorption edge between 286 to 289 eV. No detectable nitrogen features were observed in N-XANES spectra of the OM aggregate, probably due to low concentration of nitrogen, while matrix showed a small peak at 401.0 eV that is tentatively assigned to amines^16,17^ (Fig. 2f). Amines have peaks at 287.7–288.8 eV in C-XANES^16^, and this is consistent with the C-XANES spectrum of the matrix that showed small peaks at ~288.5 eV, although majority of 288.5 eV absorption is due to carboxyl/ester [(C=O)O] (Fig. 2e). The 401.0 eV peak could be atmospheric N_2_ which was either trapped in the inorganic phase or generated during X-ray exposure^17^, but high δ^15^N (shown below) in the matrix area indicate the presence of indigenous nitrogen compounds. The O-XANES of the matrix showed a peak at 531 eV indicating C=O, while that of the OM aggregate did not (Fig. S4), and it is consistent with the C-XANES.

**Figure 3:**
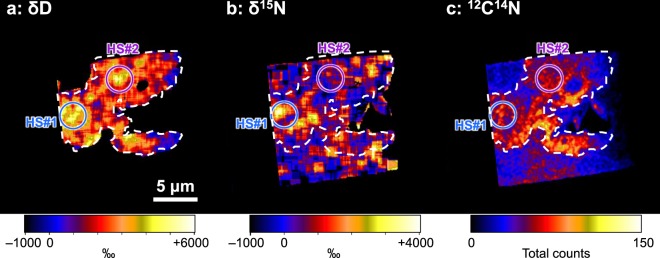
NanoSIMS isotope images of the FIB section containing the organic matter (OM) aggregate in the Zag clast (same section as for Fig. 2). (**a**) δD image, (**b**) δ^15^N image and (**c**) ^12^C^14^N ratio image. The OM aggregate is indicated by the white dotted line. Isotopic hot spots are indicated by circles.

Figure 3 shows NanoSIMS δD, δ^15^N and ^12^C^14^N images of the FIB section containing the OM aggregate (same section shown in Fig. 2). Hydrogen, nitrogen and carbon isotopic and elemental ratios of the OM aggregate and surrounding matrix are summarized in Table 1. The OM aggregate had a large δD and δ^15^N anomaly; δD = +2,370 ± 74‰ and δ^15^N = +696 ± 100‰ on average. The δD of the OM aggregate was also similar to the value of IOM from CR chondrites and the Bells meteorite, but the δ^15^N was much higher than the value of these^18^ (Supplementary Fig. 6) and rather among the values of cometary materials (δ^15^N ~ 400–1200‰^19^). The δ^13^C value was −43 ± 20‰ that was broadly consistent with the values of IOM from CR chondrites and the Bells meteorite (an unusual CM2 chondrite)^18^ within analytical error. Two isotopic hot spots were observed; one is D- and ^15^N-rich (δD = +4,200 ± 550‰ and δ^15^N = +3,413 ± 1,070‰), and the other is D-rich (δD = +4,500 ± 900‰) and less ^15^N-rich (+724 ± 780‰) (Fig. 3). These enrichments of the heavy isotopes of H and N suggest that the OM or its precursor(s) formed by low-temperature chemistry in molecular clouds or the outer protosolar disk^20^. The origin of the isotope heterogeneities (hot spots) in the OM aggregate in the Zag clast is puzzling since no molecular heterogeneity was observed between the hot spots and the average OM area (Fig. 2e,f). The absence of a correlation between chemistry and various isotopic compositions is also observed in chondritic OM^21^.

**Table 1:** Hydrogen, nitrogen and carbon isotopic and elemental ratios of organic matter (OM) aggregate and matrix of the Zag clast measured by NanoSIMS.

	δD ‰	δ^15^N ‰	δ^13^C ‰	H/C	N/C	O/C^a^
OM aggregate	2,370 ± 74	696 ± 100	−43 ± 20	0.6 ± 0.1	0.022 ± 0.004	(0.06–0.07) ± 0.02
Hot spot #1	4,200 ± 550	3,413 ± 1,070		0.4 ± 0.1	0.032 ± 0.006	
Hot spot #2	4,500 ± 900	724 ± 780				
Matrix	—	301 ± 98	10 ± 41		0.036 ± 0.007	~1.4–1.6
CI IOM^b^	972–978	30.7–31.9	−17.1 to −17.0	0.67–0.72	0.035–0.035	0.18–0.15
CM IOM (except Bells)^b^	639–893	−8.5 to +7.5	−18.9 to 17.1	0.52–0.70	0.026–0.037	0.11–0.23
Bells IOM (Anomalous CM)^b^	3283	415	−34.2	0.63	0.034	0.21
CR IOM^b^	2,619–3,527	162–309	−26.6 to −20.3	0.69–0.81	0.032–0.044	0.11–0.22
Ordinary chondrite IOM^b^	1,917–6,181	−39 to +36	−23.7 to −10.4	0.16–0.48	<0.019	0.14–0.29
Tagish Lake IOM^c^	596 to 1,844	53 to 73	−14.7 to −13.3	0.34–0.72	0.041–0.043	0.13–0.26
Comets	up to ~2,200^d^	~400–1200^e^			0.035 ± 0.011^f^	

^a^The upper limits estimated by C,N,O *K*-edge X-ray absorption spectra. Note that N/C ratio is too low to be estimated by XANES.

^b^Insoluble organic matter (IOM) data from Alexander *et al*.^18^.

^c^IOM data from Herd *et al*.^42^ and Alexander *et al*.^41^.

^d^Cometary water data from Altwegg *et al*.^35^ and references therein.

^e^Data from Marty^19^ and references therein.

^f^Data from Fray *et al*.^29^.

N-XANES spectra and NanoSIMS ^12^C^14^N images of the Zag clast FIB section showed a relatively higher concentration of nitrogen in the matrix region compared to that in the aggregate. An estimation by NanoSIMS for N/C elemental ratio of matrix was 0.036 ± 0.007 while N/C ratio of OM aggregate was 0.022 ± 0.004. The majority of carbon in the matrix comes from carbonates, therefore the N/C_OM_ ratio of the matrix would have been higher with a lower C_OM_ abundance.

## Discussion

The large, micrometer-sized OM grains/aggregates are abundant in the Zag clast but are rare in other meteorites - a very few are known in CR chondrites^22,23^. The C-XANES spectrum of the OM aggregate does not resemble IOM in primitive CI/CM/CR chondrites that shows C=O at ~286.5 eV, (C=O)O at ~288.5 eV and sometime aliphatic carbon at ~287.5 eV^14^. Even the C-XANES spectra of the IOM from thermally-metamorphosed chondrites (e.g., CV and CO chondrites and ordinary chondrites) have a 288.5 eV peak, in addition to 1s-σ* exciton at 291.7 eV indicating graphene structures^15^, this is not the case for the OM aggregate. The H/C and N/C ratios of the OM aggregate obtained by NanoSIMS (Table 1) are falls between these of CI/CM/CR chondrite-IOM cluster and CV/CO and ordinary chondrite-IOM cluster^18^ (Supplementary Fig. 6). An estimation for the O/C elemental ratio of the OM aggregate from C,N,O X-ray absorption spectra is 0.06 to 0.07 (uncertainty is ±0.02), that is lower than IOM extracted from CV, CO and ordinary chondrites^18^. Note that the O/C ratio is an upper limit due to the possibility of some contribution from silicates. The high δD has been seen in IOM from CR chondrites, Bells meteorite (an anomalous CM chondrite) and Ordinary chondrites, and high δ^15^N has only been observed in CRs and Bells^18^. However, IOM from CRs and Bells are not very aromatic and are rich in oxygen^24^. Thus, H- and N isotopic compositions also indicate uniqueness of the OM aggregate.

On the other hand, C-XANES spectra of “aromatic” nanoglobules in chondrites reported by De Gregorio *et al*.^21^ are similar to the OM aggregate in the Zag clast. In their study, some aromatic nanoglobules tend to have higher δ^15^N values than IOM-like nanoglobules, although the correlation between molecular structure and δ^15^N was rather ambiguous^21^. The OM aggregate has isotopic heterogeneities without molecular structure heterogeneities, and it indicates that the OM aggregate consists of materials with different origins but which subsequently experienced similar chemical evolution pathways. Note that we also found a globular OM grain in the Zag clast (Supplementary Fig. 1) but larger (~5 μm) than typical nanoglobules (<1 μm).

The OM aggregate in the Zag clast studied here is somewhat similar to ultracarbonaceous Antarctic micrometeorites (UCAMM) that are considered as cometary materials^25^, with respect to the size and the high concentrations of heavy isotopes (D and ^15^N), although UCAMMs are anhydrous and not always D- and ^15^N-rich^26^. However, C- and N-XANES spectra of UCAMM showed absorption edges for O- and N-bearing functional groups, e.g., C=O, (C=O)O, C=N, and NHx(C=O)^26^, that is not the case for the OM aggregate in the Zag clast. Cometary OM (CHON particles from comet Halley and returned samples from comet 81 P/Wild 2) has higher H, N and O contents^27,28^, compared to the OM aggregates. Although, cometary particles from 67 P/Churyumov-Gerasimenko analyzed by the COSIMA instrument on board the Rosetta mission have lower N contents, i.e., N/C = 0.035 ± 0.011^29^. The C-XANES spectra of comet 81 P/Wild2 particles, as well as anhydrous and hydrated chondritic interplanetary dust particles and chondritic micrometeorites (some of which probably originated from comets) also show O-bearing functional groups (e.g., C=O at ~286.5 eV, (C=O)O at ~288.5 eV)^27,30^.

The surrounding matrix contains N-rich compounds. These N-bearing compounds would not share the same origin as the OM aggregate since the δ^15^N value of the OM is ~700 ± 100‰ while the matrix is ~300 ± 100‰. IOM in carbonaceous chondrites is known to release ammonia up to 10 µg/mg via hydrothermal processing at 300–400 °C, but the δ^15^N of the released fractions are higher than that of the original IOM^31^. Alternatively, the differences in δ^15^N value would be attributed to isotope fractionation due to release of N-bearing components from OM aggregate during alteration. In some thermally metamorphosed chondrites (e.g., Isheyevo meteorite), ^15^N-rich component is associated with thermally resistant organic moieties^32^. In this case, ^15^N-rich compound could remain in the OM aggregate.

The high D/H and ^15^N/^14^N ratios suggest that the precursor molecules of OM aggregate originated in a very cold environment such as the interstellar medium or the outer region of the solar nebula. The OM aggregate could have been formed from small precursor molecules from protoplanetary disc or interstellar medium such as formaldehyde, during aqueous alteration after incorporated into planetesimals, proposed by Cody *et al*.^33^. Relatively high temperature process (~200 °C) and/or additional ingredient such as ammonia could enhance aromatic units^34^. In the case of carbonaceous chondrites, significant decreases in D/H ratio of OM are suggested to be accompanied by aqueous alteration mostly due to D-H exchange with D-poor water, e.g., the δD value of OM in the most aqueously altered CI chondrites is ~+970–980‰ in contrast to the high δD values of the least altered carbonaceous chondritic OM (up to ~+3,500‰)^18^. If this is the case of the Zag clast, the D/H ratio of the OM is expected to be reduced during the heavy aqueous alteration reflected in the mineralogy of the clast, i.e., CI chondrite like compositions^12^. Therefore, D-rich water is required to maintain the high D/H ratio (δD ~+2,400‰) in the OM aggregate, such as the water in the outer Solar System bodies, e.g., some comets and Enceladus which have D/H ratios up to ~5 × 10^−4^ (δD ~+2,200‰)^35^. The fact that the OM aggregate maintains high D/H ratio after heavy aqueous alteration indicates that the water should have high D/H at the time of alteration. Thus, the clast was most likely originated from the outer region of the Solar System. However, it should be noted that if OM precursors have had much higher D/H ratio than current value, e.g, up to D/H ~ 0.5 for interstellar formaldehyde^36^, the reaction with lower D/H water would have decreased the D/H ration of OM to the current value. The morphology of the OM aggregate indicates that smaller OM particles were concentrated by fluids, while simultaneously, the molecular structure of the OM was modified by aqueous alteration. This could also explain the isotopic hot spots. The low temperature and extended period of the aqueous event could have decreased substituted functional groups of the OM structure likely via aromatization, oxidation and decarboxylation during hydrothermal alteration. The C-XANES of OM in the surrounding matrix shows higher oxygen containing functional groups compared to the OM aggregates (Fig. 1e). This is probably because hydrophobic nature of the OM aggregate likely tends to form aggregate besides hydrophilic nature of O-rich OM tend to be diffused in the matrix during aqueous activities.

The O-isotopic compositions of the Zag clast are clearly distinguished from that of any known chondrite groups. The isotopic evidences indicate that the parent body of the clast consisted of primitive materials However, the mineralogical, and organic molecular structural indicate the clast was subjected to aqueous alteration, and it discriminates the clast from comets and related materials. We propose that possible candidates of the clast parent body are D/P type asteroids, trans-Neptunian objects (TNOs), or comets due to the following reasons.

The dynamical evolution of the giant-planet orbits leads to the insertion of primitive TNOs into the outer main belt asteroid region as D/P type asteroids^37,38^. Some of these orbits could have been scattered to the inner main belt region, and contaminated the probable parent body of the H chondrites, asteroid 6/Hebe at 2.4 AU^39^. The nature of D/P type asteroids are not well known. The only material potentially identified as having a D/P type asteroid origin is the Tagish Lake meteorite^40^. The Tagish Lake meteorite is dominated by phyllosilicates with locally abundant carbonates, sulfides and magnetite, and is mineralogically very similar to the Zag clast. One difference is that Tagish Lake contains no halite. C-XANES spectra indicate that the Tagish Lake IOM has more O-bearing compounds^41^ compared to the OM aggregate in the Zag clast. IOM in the Tagish Lake meteorite is D- and ^15^N-rich (δD = +596 to 1,844‰ and δ^15^N = +53 to 73‰)^42^, but the D/H ratio of water in the Tagish Lake meteorite is estimated to be lower than the value of the terrestrial water^43^. Bulk oxygen isotopic compositions of the Tagish Lake meteorite are δ^18^O = +18.0 to 19.0‰ and δ^17^O = +8.3 to 9.2‰ (analytical uncertainties are 0.1)^44^, that are also inconsistent with the Zag clast O-isotopic compositions. The above evidences indicate that the Zag clast does not share the same (or similar) origin with the Tagish Lake meteorite. However, we must be careful not to jump to a conclusion that the Tagish Lake meteorite could be a representative sample of D/P type asteroids, as there could be large sampling biases due to physical processes during delivery to Earth. It further implies a possibility that the origin of the clast could be a comet. Although comets are traditionally considered to be anhydrous, some mineralogical evidences for aqueous activity on Comet 81P/Wild 2 were reported^45,46^.

The OM aggregate and the clast provide a novel evidence that long-lasting low-temperature aqueous alteration in isotopically primitive body(s). The isotopic and organic structure of the OM aggregate and the clast clearly show that the clast is unique among known chondrite group and cometary materials. We propose that the clast is possibly sample from D/P asteroids or TNOs. Currently, the information of these bodies is highly limited based on ground based and/or spacecraft observation. Combined with further study of such clasts as well as future missions to D/P asteroids will provide insight into primitive volatile-rich solar system small bodies.

## Methods

### Oxygen isotope analysis

Oxygen isotope analysis was undertaken by laser-assisted fluorination^47^ on a 2 mg aliquot of the clast drawn from a ~20 mg homogenized bulk powder. Note that the aliquot used for the O-isotope analysis was taken from the same clast but different aliquot which used in the rest of analyses. We could not conduct replicate analysis due to the limitation of the sample amount. Thus, we have taken the average of the CR2 and NWA 2086 errors as for the precision of the Zag clast analysis. The 1σ error of δ^17^O ‰, δ^18^O ‰, and ∆^17^O ‰ are 0.13, 0.17, and 0.042, respectively.

### Sample preparation using a focused ion beam (FIB)

The OM aggregate was selected from a polished thin section of the xenolithic clast in the Zag meteorite using imaging from a JEOL 7600F field emission gun scanning electron microscope (FEG-SEM) at NASA/JSC. Approximately 100 nm-thick sections were subsampled from the OM aggregate in the Zag clast using a Quanta 3d FEG focused ion beam (FIB) instrument at NASA/JSC.

### Scanning Transmission X-ray Microscopy (STXM)

Carbon, nitrogen and oxygen X-ray absorption near edge structure (C,N,O-XANES) micro-spectroscopy was performed using the scanning transmission X-ray microscopes (STXM) at BL-13A of the Photon Factory, High Energy Accelerator Research Organization (KEK)^48,49^. The carbon map was obtained by acquiring pairs of images below and on the carbon *K*-edge, at 280 and 292 eV, respectively, and taking the –ln(*I*_292_/*I*_280_) for each pixel. The C-XANES spectra were acquired with the energy step sizes (Δ*E*) of 0.1 eV in 283–295.5 eV region, 0.5 eV in 280–283 eV and 295.5–301.0 eV regions, and 1 eV in 301–310 eV region. For N-XANES, Δ*E* was 0.2 eV in 395–406 eV region, 0.5 eV in 385–395 eV and 406–410 eV regions, and 2 eV in 410–430 eV region. For O-XANES, Δ*E* was 0.2 eV in 530–540 eV region, 1 eV in 520–530 eV and 540–560 eV regions, and 2 eV in 560–580 eV region. The acquisition time per energy step was 5 to 10 ms. STXM-XANES data analysis was performed using the software aXis2000 (http://unicorn.mcmaster.ca/aXis2000.html). The O/C elemental ratio of the OM aggregate was calculated from C,N,O X-ray absorption spectra, using the method reported in Cody *et al*.^27^, the uncertainty due to fitting is ± 0.02.

### NanoSIMS ion microprobe

Hydrogen, carbon, and nitrogen isotope imaging measurements of the Zag clast FIB section were carried out with the JAMSTEC NanoSIMS 50 L. Detailed measurement conditions are described elsewhere^50,51^. Briefly, a focused Cs^+^ primary ion beam of 0.8 to 4 pA was rastered over 25 µm × 25 µm areas on the sample and a standard material (1-hydroxybenzotriazole (HOBT) hydrate; C_6_H_5_N_3_O·*x*H_2_O, calculated as *x* = 1). The spatial resolution was estimated to be ~100 nm for C and N isotope images, and ~200 nm for H isotope image. Each run repeatedly scanned (10 to 20 times) over the same area. Individual images consist of 256 × 256 pixels with acquisition time of 6,000 µs/pixel (393 sec/frame) for C and N isotope images, and of 5,000 µs/pixel (328 sec/frame) for H isotope image. Each measurement was started after stabilization of the secondary ion intensities following a pre-sputtering procedure of approximately 1–3 min. The sample was coated with a 10 nm Au thin film to mitigate electrostatic charge on the surface. During the analysis, the mass peaks were centered automatically every 5 cycles. The final isotope images were generated from regions that have statistically enough counts. Note that hydrogen signals were very low in the matrix region, likely due to small amount of phyllosilicate in the matrix, and/or less ionization efficiency of hydrogen in phyllosilicates compared with that in OM under the Cs^+^ primary ion bombardment.

The OM regions have been chosen by distributions of ^12^C within a section applying 10% threshold of total ^12^C ion counts. Thus, minerals and OM regions was distinguished by above method with a spatial resolution of ~100 nm.

The N/C ratios were calculated using HOBT hydrate as an elemental abundance standard. Our estimation of the uncertainty from the calibration with single standard would be 10–20%, based on the work by Alleon *et al*.^52^.

### Possible D-enrichment during sample preparation and analyses

D/H enhancement up to 1,000‰ could be produced by exposure of an organic sample to the electron beam^53,54^. Although our sample have never been exposed to such strong irradiation, the δD of the OM aggregate is over 2,000‰ with hot spots over 4,000‰. Even if the D/H ratio is enhanced by the irradiation, the OM aggregate still has significant D-enrichment. Note that one of the samples prepared and analyzed by the same methods did not show such high δD value^11^.

## Supplementary information

Supplementary Info

## Data Availability

Correspondence and requests for materials should be addressed to Y.K.
