# Supplementary material for: A novel organic-rich meteoritic clast from the outer solar system
Source: Sci Rep. 2019 Feb 28;9:3169. doi: 10.1038/s41598-019-39357-1 (PMC6395772; doi:10.1038/s41598-019-39357-1)
Supplement: Supplementary file 1 — Supplementary Info [file 41598_2019_39357_MOESM1_ESM.pdf]

Supplementary Information

A novel organic-rich meteoritic clast from the outer solar system

Yoko Kebukawa, Motoo Ito, Michael E. Zolensky, Richard C. Greenwood, Zia Rahman,
Hiroki Suga, Aiko Nakato, Queenie H. S. Chan, Marc Fries, Yasuo Takeichi, Yoshio Takahashi,
Kazuhiko Mase, and Kensei Kobayashi

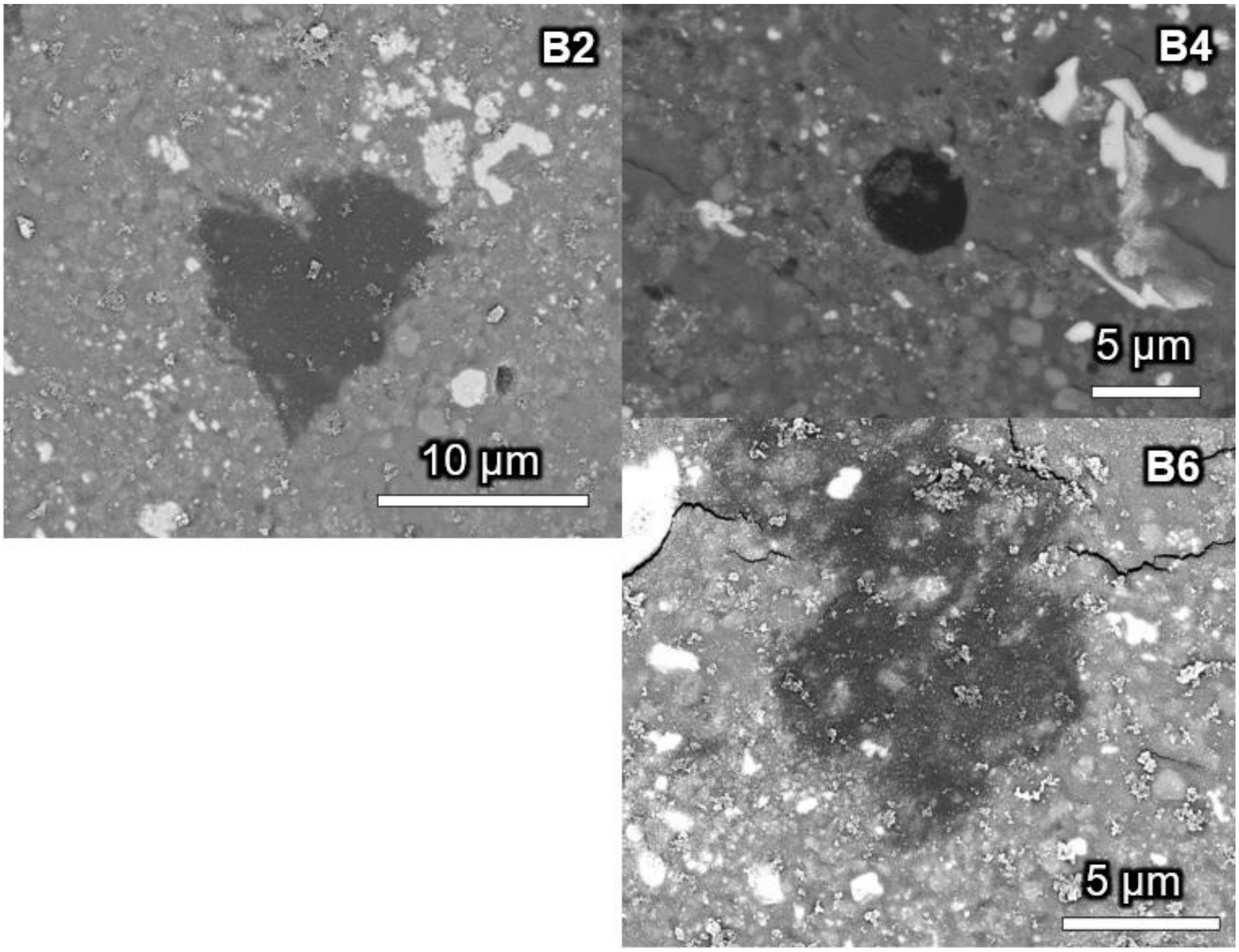

Supplementary Figure 1: **Backscattered electron (BSE) images of organic grains/aggregates (black) in the clast in the Zag clast.**

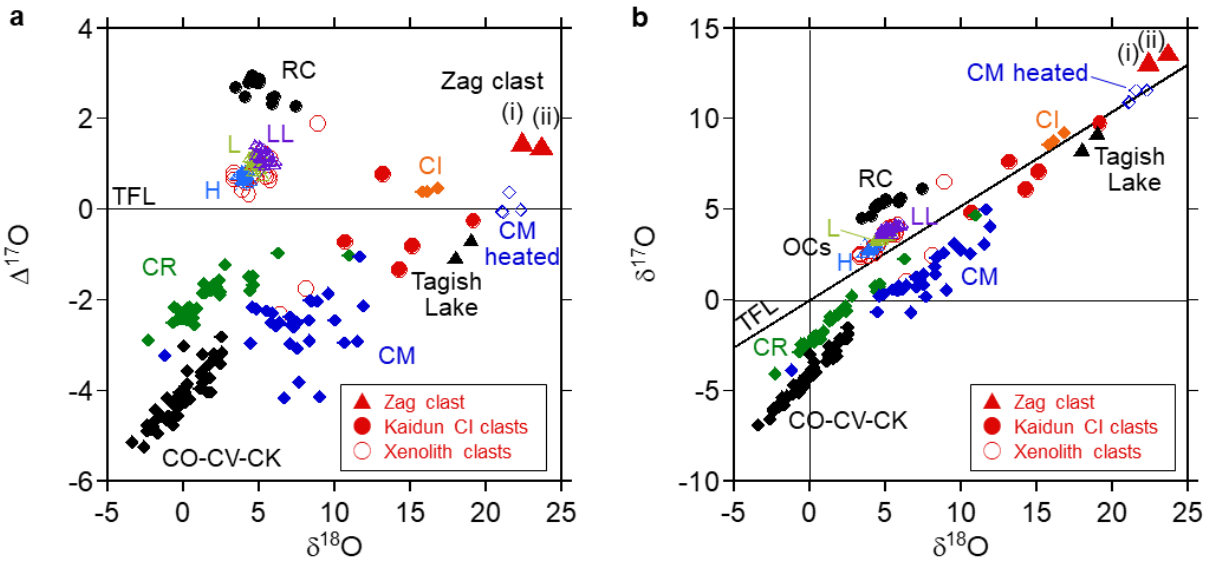

Supplementary Figure 2: **Oxygen isotope composition of the Zag clast shown in relation to various chondrite groups and carbonaceous clasts in other meteorites.** (a) $\Delta^{17}O$ vs. $\delta^{18}O$ plot, and (b) $\delta^{17}O$ vs. $\delta^{18}O$ plot. The clast in Zag meteorite is distinguished from any other chondrites, CI clasts in Kaidun meteorite nor xenolith clasts from various meteorites. Ordinary chondrite (H, L, LL) data from Clayton et al. (1991)[1], CI, CM chondrite data from Clayton and Mayeda (1999)[2], CR chondrite data from Clayton and Mayeda (1999)[2] and Schrader et al. (2011, 2014)[3,4], CV and CK chondrite data from Greenwood et al. (2010)[5], CO chondrite data from Alexander et al., (2018)[6], Tagish Lake data from Brown et al. (2000)[7]. CI clasts in Kaidun meteorite and xenolith clasts data from Clayton et al. (1991)[1]and Clayton and Mayeda (1999)[2]. Zag clast (i): this study, Zag clast (ii): analysis of the same clast reported by Zolensky et al. (2003)[8]. TFL: terrestrial fractionation line. The errors are smaller than the symbol sizes.

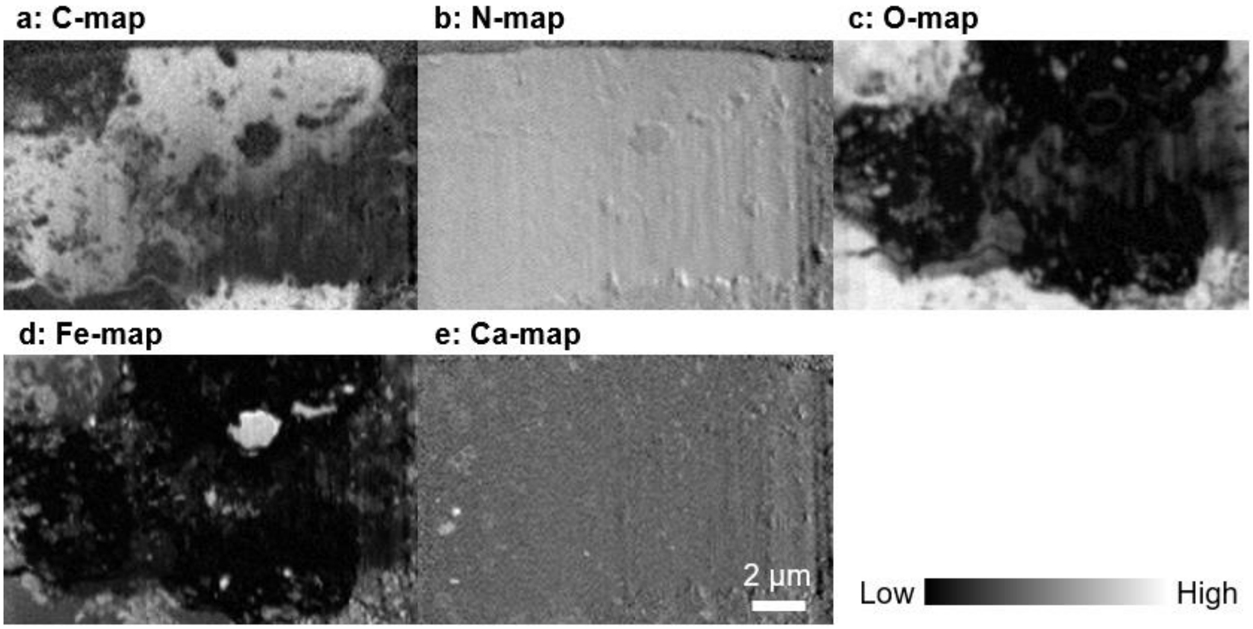

Supplementary Figure 3: **STXM elemental map of the FIB section including the OM aggregates in the Zag clast.** (a) C-map: $–\ln(I_{292}/I_{280})$, (b) N-map: $–\ln(I_{405}/I_{395})$, (c) O-map: $–\ln(I_{539}/I_{525})$, (d) Fe-map: $–\ln(I_{709}/I_{705})$, and (e) Ca-map: $–\ln(I_{349}/I_{345})$. The C-map indicates the OM aggregate. The matrix region is dominated in oxygen and contains some iron, likely associated phyllosilicates and carbonates.

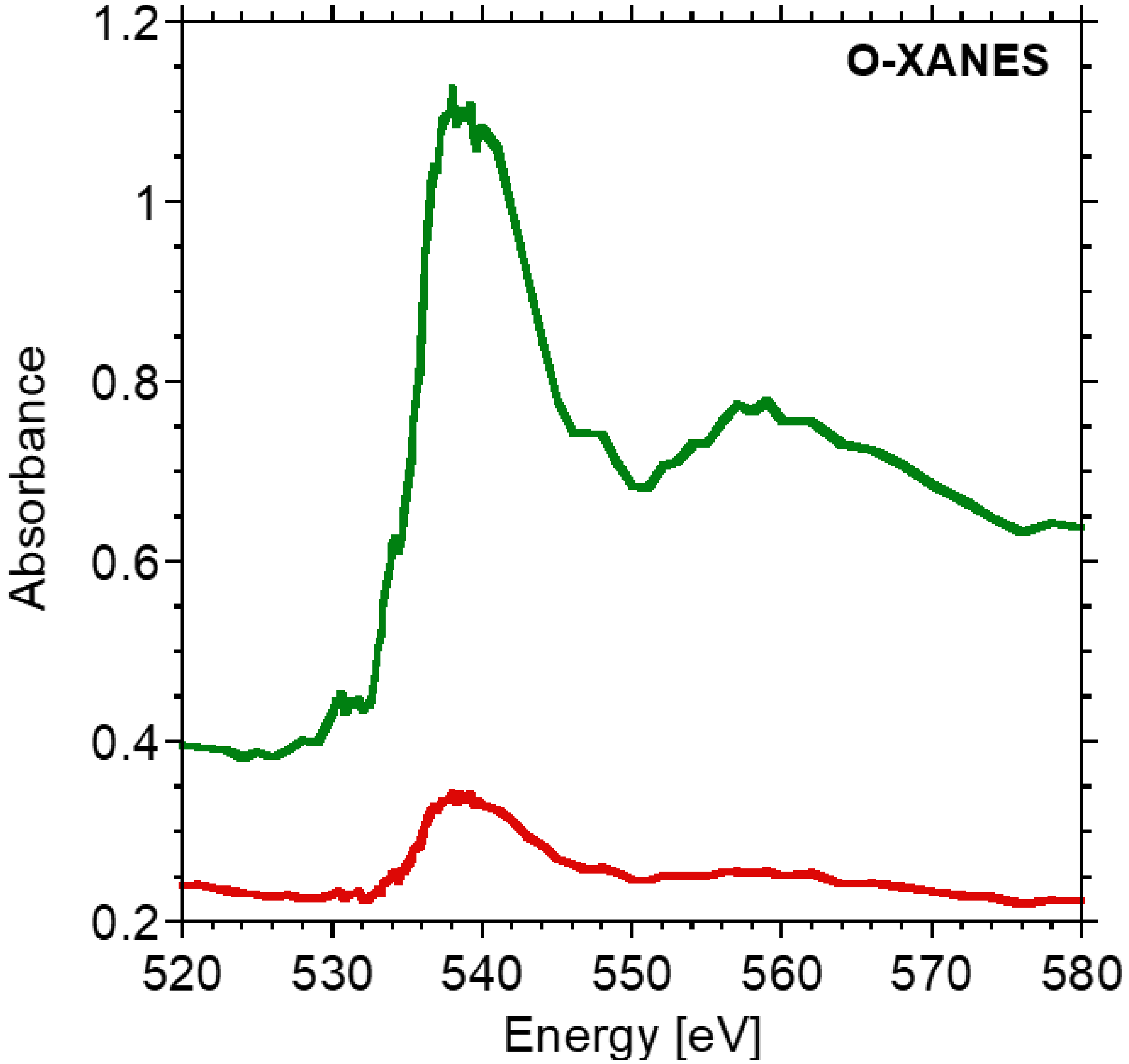

Supplementary Figure 4: **O-XANES spectra of the Zag clast.** The OM aggregate is shown in red, and surrounding matrix is in green.

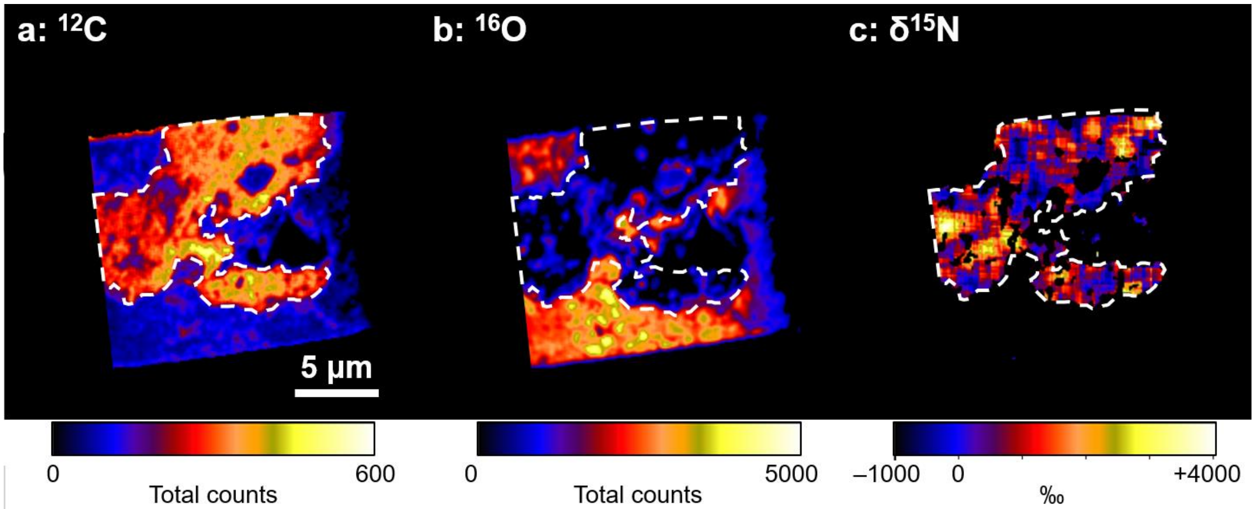

Supplementary Figure 5: **NanoSIMS isotope images of the FIB section containing the organic matter (OM) aggregate in the Zag clast.** (a) ^{12}C image, (b) ^{16}O image and (c) Selected $\delta^{15}N$ image of OM area.

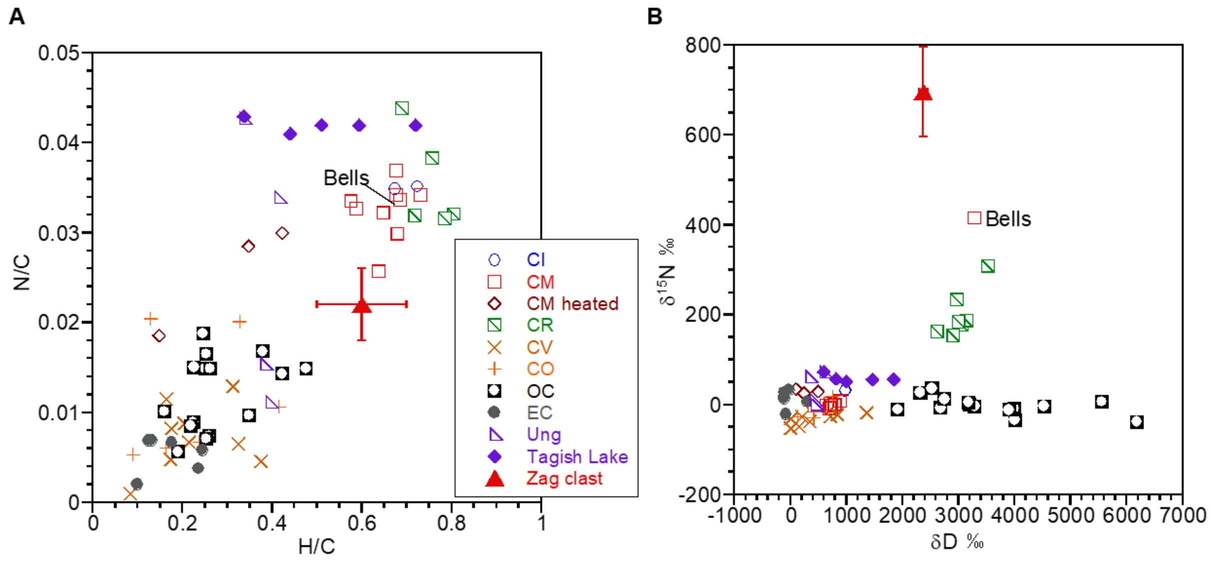

Supplementary Figure 6: **Elemental and isotopic compositions of the Zag clast OM shown in relation to insoluble organic matter from various chondrite groups.** (a) N/C vs. H/C ratios (atomic), and (b) δD vs. δ^{15}N. CI, CM, CR, CV, CO chondrites, ordinary chondrites (OC) enstatite chondrites (EC), and ungrouped chondrites (Ung) data from Alexander et al.[9], and Tagish Lake data from Herd et al.[10].

References

1 Clayton, R. N., Mayeda, T. K., Goswami, J. & Olsen, E. J. Oxygen isotope studies of ordinary chondrites. *Geochimica et Cosmochimica Acta* **55**, 2317-2337 (1991).

2 Clayton, R. N. & Mayeda, T. K. Oxygen isotope studies of carbonaceous chondrites. *Geochimica et Cosmochimica Acta* **63**, 2089-2104 (1999).

3 Schrader, D. L., Franchi, I. A., Connolly, H. C., Jr., Greenwood, R. C., Lauretta, D. S. & Gibson, J. M. The formation and alteration of the Renazzo-like carbonaceous chondrites I: Implications of bulk-oxygen isotopic composition. *Geochimica et Cosmochimica Acta* **75**, 308-325 (2011).

4 Schrader, D. L., Davidson, J., Greenwood, R. C., Franchi, I. A. & Gibson, J. M. A water–ice rich minor body from the early Solar System: The CR chondrite parent asteroid. *Earth and Planetary Science Letters* **407**, 48-60 (2014).

5 Greenwood, R. C., Franchi, I. A., Kearsley, A. T. & Alard, O. The relationship between CK and CV chondrites. *Geochimica et Cosmochimica Acta* **74**, 1684-1705 (2010).

6 Alexander, C. M. O. D., Greenwood, R. C., Bowden, R., Gibson, J. M., Howard, K. T. & Franchi, I. A. A mutli-technique search for the most primitive CO chondrites. *Geochimica et Cosmochimica Acta* **221**, 406-420 (2018).

7 Brown, P. G., Hildebrand, A. R., Zolensky, M. E., Grady, M., Clayton, R. N., Mayeda, T. K., Tagliaferri, E., Spalding, R., MacRae, N. D., Hoffman, E. L., Mittlefehldt, D. W., Wacker, J. F., Bird, J. A., Campbell, M. D., Carpenter, R., Gingerich, H., Glatiotis, M., Greiner, E., Mazur, M. J., McCausland, P. J. A., Plotkin, H. & Mazur, T. R. The fall, recovery, orbit, and composition of the Tagish Lake meteorite: A new type of carbonaceous chondrite. *Science* **290**, 320-325 (2000).

8 Zolensky, M., Clayton, R., Mayeda, T., Chokai, J. & Norton, O. Carbonaceous chondrite clasts in the halite-bearing H5 chondrite Zag. *Meteoritics and Planetary Science Supplement* **38** (2003).

9 Alexander, C. M. O. D., Fogel, M., Yabuta, H. & Cody, G. D. The origin and evolution of chondrites recorded in the elemental and isotopic compositions of their macromolecular organic matter. *Geochim. Cosmochim. Acta* **71**, 4380-4403 (2007).

10 Herd, C. D. K., Blinova, A., Simkus, D. N., Huang, Y., Tarozo, R., Alexander, C. M. O. D., Gyngard, F., Nittler, L. R., Cody, G. D., Fogel, M. L., Kebukawa, Y., Kilcoyne, A. L. D., Hilts, R. W., Slater, G. F., Glavin, D. P., Dworkin, J. P., Callahan, M. P., Elsila, J. E., De Gregorio, B. T. & Stroud, R. M. Origin and evolution of prebiotic organic matter as inferred from the Tagish Lake meteorite. *Science* **332**, 1304-1307 (2011).
